# Development and Validation of the Computerised Adaptive Beat Alignment Test (CA-BAT)

**DOI:** 10.1038/s41598-018-30318-8

**Published:** 2018-08-17

**Authors:** Peter M. C. Harrison, Daniel Müllensiefen

**Affiliations:** 1 School of Electronic Engineering and Computer Science, Queen Mary University of London, London, E1 4NS United Kingdom; 2 Department of Psychology, Goldsmiths, University of London, London, SE14 6NW United Kingdom; 3 University of Music, Drama, and Media, Hanover, Germany

## Abstract

Beat perception is increasingly being recognised as a fundamental musical ability. A number of psychometric instruments have been developed to assess this ability, but these tests do not take advantage of modern psychometric techniques, and rarely receive systematic validation. The present research addresses this gap in the literature by developing and validating a new test, the Computerised Adaptive Beat Alignment Test (CA-BAT), a variant of the Beat Alignment Test (BAT) that leverages recent advances in psychometric theory, including item response theory, adaptive testing, and automatic item generation. The test is constructed and validated in four empirical studies. The results support the reliability and validity of the CA-BAT for laboratory testing, but suggest that the test is not well-suited to online testing, owing to its reliance on fine perceptual discrimination.

## Introduction

Music listening invokes many cognitive abilities, including for example auditory scene analysis^1^, pitch and rhythm perception^2^, beat perception^3^, syntactic processing^4^, and emotion induction^5^. Though most listeners reach a baseline competence in these tasks, precise ability levels differ between individuals^6,7^. In music perception research, these individual differences help to identify and dissociate the psychological and neural bases of music perception^7,8^. In education, these individual differences help diagnose musical aptitude (a student’s capacity for future musical success) as well as forming an important aspect of trained musicianship^9,10^.

Various standardised tests of musical listening abilities have been developed over the last century, ranging from the early Measures of Musical Talents^11^ to recent measures such as the Musical Ear Training Assessment (META)^10^ and the children’s Rhythm Synchronization and Melody Discrimination tasks^12^. These measures are essential for achieving efficient and objective ability testing in research and education environments, and have supported much research into the origin, nature, and distribution of musical abilities in the human population^6,13–16^.

**Figure 1:**
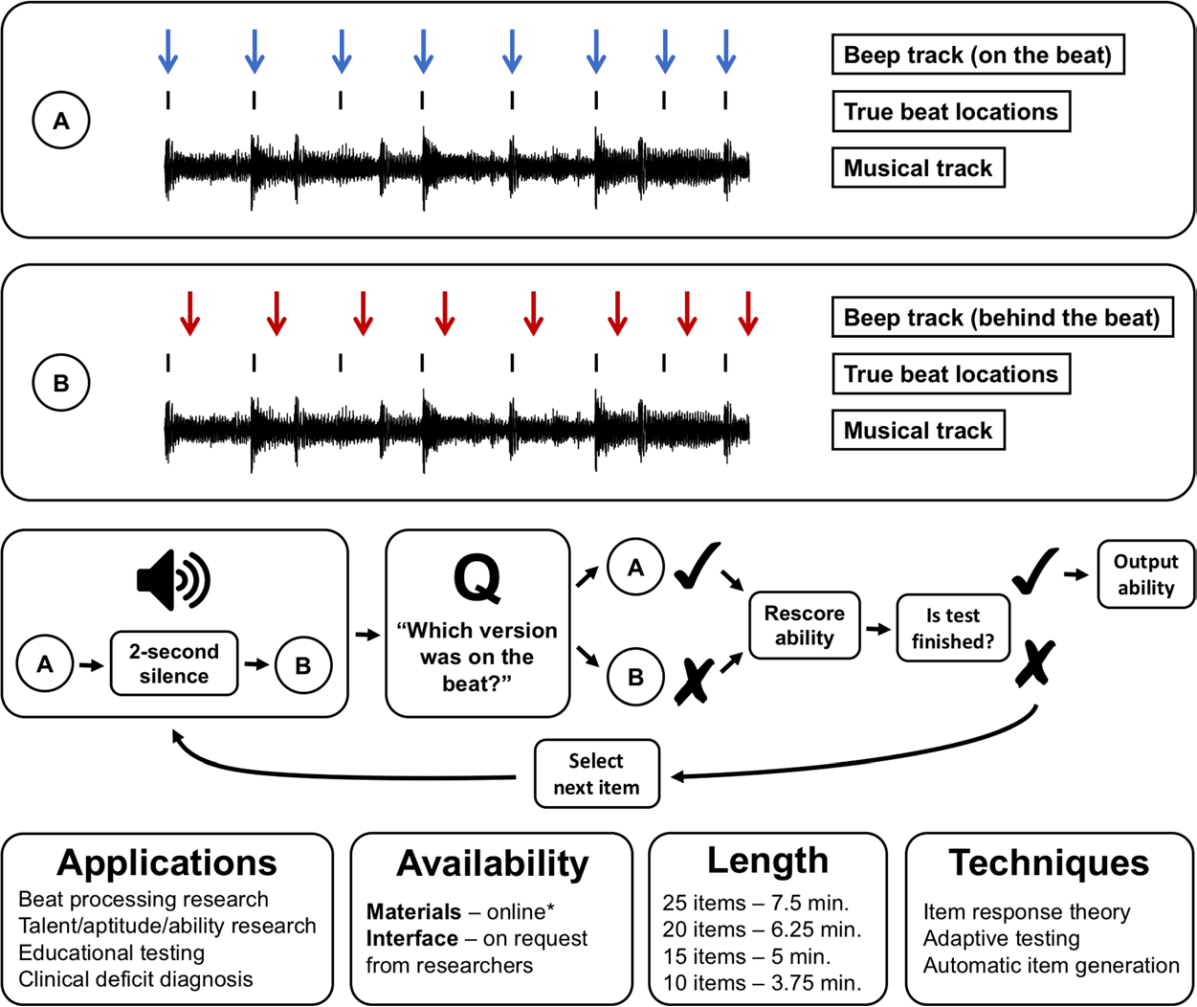
Schematic figure for the CA-BAT summarising the test’s format, potential applications, availability, length, and underlying psychometric techniques. *Test materials are available online at https://doi.org/10.5281/zenodo.1210808.

Testing techniques have improved significantly since the original Measures of Musical Talents. Advances in recording technology have substantially improved the quality of audio stimuli^17^, and the adoption of signal detection theory has addressed the response bias confounds of early tests^6,17^. Adaptive techniques have become the norm for low-level perceptual abilities such as pitch discrimination, allowing tests to adapt dynamically to participants’ abilities^18^. Some recent listening tests have also adopted modern psychometric techniques such as item response theory^19^ and automatic item generation^20^, extending the applicability of adaptive testing to more complex tasks such as melody discrimination^21^. Nonetheless, many listening ability tests still use out-dated psychometric techniques such as classical test theory and non-adaptive testing, leaving much scope for the psychometric modernisation of musical assessment^21^.

This paper describes a modern psychometric approach to beat perception, the process of inferring an underlying pulse or beat from an extract of music^3^. This perceived beat is linked to acoustic periodicities in the sound, but it is not trivially determined by these periodicities: instead beat perception seems to be a constructive process, guided by voluntary control, and influenced by enculturation^3,22,23^. The perceived beat forms the scaffold about which musical rhythms are perceived, making beat perception an indispensable part of music perception and appreciation. Beat perception also plays a crucial role in dancing and collaborative music-making^24^, with this interpersonal synchrony thought to play an important role in facilitating social bonding^25^. Beat perception is therefore recognised as a fundamental musical ability^3,24^, and has attracted a broad range of research from psychological, neuroscientific, and clinical perspectives^6,26,27^.

Research into beat perception has been supported by the development of several beat perception tests, including the Beat Alignment Test (BAT)^28^, the Adaptive Beat Alignment Test (A-BAT)^29^, the Beat Perception Test from the Goldsmiths Musical Sophistication Index (Gold-MSI)^6^, the Harvard Beat Assessment Test (H-BAT)^30^, and the Battery for the Assessment of Auditory Sensorimotor and Timing Abilities (BAASTA)^31^. Successful applications of these tests include identifying auditory and motor areas involved in beat perception and rhythmic reproduction^26^, investigating how beat perception abilities relate to other musical abilities and behaviours^6^, and predicting which Parkinson’s patients will benefit from rhythmic auditory stimulation therapy^27^.

These instruments provide a useful variety of measures covering many behaviours related to beat perception, such as tapping to the beat in a piece of music, detecting misalignment between a metronome and a musical extract, and detecting differences in duration between successive sounds. This variety is important for developing a comprehensive picture of the different psychological mechanisms involved in beat perception. However, this proliferation of measures comes at the expense of lessened development time for each measure. One result is that current beat perception tests do not fully exploit modern psychometric techniques such as item response theory^19^ and adaptive testing^32^. A second result is a relative paucity of systematic validation: for example, test-retest reliability, an indispensable validation measure, is not reported for the BAT^28^, A-BAT^29^, or H-BAT^30^ (though see ref.^33^ for a test-retest validation of the BAASTA).

This paper takes a complementary approach: we focus on one beat perception paradigm, and use it to construct a psychometrically sophisticated and well-validated test termed the Computerised Adaptive Beat Alignment Test (CA-BAT; Fig. 1). This test probes a listener’s beat perception ability using the beat alignment paradigm of ref.^28^, where the listener tries to detect misalignment between a metronome and a musical extract. The new test uses realistic music and employs state-of-the art psychometric techniques. Item response theory provides principled item selection and sophisticated reliability quantification^19^; item response modelling links stimulus features to item difficulty, reinforcing construct validity^34^; adaptive testing tailors the difficulty level to the participant, helping the test perform well across the ability spectrum^32^; automatic item generation provides an effectively unlimited bank of computationally generated items^20^. We intend for the CA-BAT to provide a useful beat perception measure for future research, as well as providing a model for the subsequent development of future listening tests.

## Psychometric Background

This section introduces three modern psychometric techniques used in the CA-BAT: item response theory, computerised adaptive testing, and automatic item generation.

### Item response theory

Item response theory (IRT) distinguishes itself from classical test theory in its item-level rather than test-level focus (see ref.^19^ for an overview). Most IRT models are special cases of the four-parameter logistic model, defined as

1 $$P({X}_{j}\mathrm{=1}|\theta ,{a}_{j},{b}_{j},{c}_{j},{d}_{j})={c}_{j}+({d}_{j}-{c}_{j})\frac{\exp [{a}_{j}(\theta -{b}_{j})]}{1+\exp [{a}_{j}(\theta -{b}_{j})]}$$

with *X*_*j*_ denoting the scored response of the test-taker to item *j* (1 = correct, 0 = incorrect), *θ* being the *ability* parameter for the test-taker, and *a*_*j*_, *b*_*j*_, *c*_*j*_, and *d*_*j*_ being the item parameters for item *j*, termed *discrimination*, *difficulty*, *guessing*, and *inattention* respectively^35^.

Each item parameter captures a distinct item characteristic. High discrimination means that the item discriminates well between test-takers of different abilities. High difficulty means that a high ability is required to answer the item correctly. A high guessing parameter means that the item is easy to guess correctly, whereas a low inattention parameter means that high-ability participants still sometimes deliver incorrect answers.

Under IRT, test-taker ability is measured on the same scale as item difficulty. This scale is usually scaled and centred so that the distribution of abilities in the test-taker population has mean 0 and standard deviation 1.

Item parameters must be estimated before participant ability can be scored. This is typically done as part of the test construction phase. This parameter calibration process can be one of the most expensive parts of IRT test construction, since many participants (hundreds to thousands) are often required to achieve reliable parameter estimates^19^.

IRT possesses several important advantages as compared to classical test theory. Primary among these is the ability to compare test-taker scores even when the test-takers answer different test items. This means that tests can easily be shortened or lengthened without compromising score comparability, and it is an important prerequisite for adaptive testing.

A second advantage is a more sophisticated treatment of reliability. In classical test theory, reliability is a function of a test and a test-taker population; it is difficult to generalise reliability estimates from one population to another, and difficult to generalise from one test configuration to another. In IRT, reliability is instead a function of the items administered and the test-taker’s ability level, and can be computed solely from a participant’s response pattern and the item parameter estimates. The ability to tailor reliability estimates to an individual participant and their testing configuration makes for a much more sophisticated reliability estimator than in classical test theory.

IRT is rarely used in musical psychology research, but it seems that music psychology stands to benefit from its incorporation^21^. So far the only published beat perception test to use IRT is the Gold-MSI Beat Perception Test^6^.

### Adaptive testing

In adaptive testing, item selection adapts to the test-taker’s previous responses (see ref.^32^ for an overview). Typically the aim is to maximise the amount of information that each successive item gives about the test-taker’s true ability level, meaning in practice that poorly performing candidates receive easier items, and better performing candidates receive harder items.

Several approaches exist for adaptive testing. The simplest approaches, such as the staircase method^36^ and the maximum-likelihood procedure^37^, are quick to calibrate but have limited flexibility, being best suited to simple psychophysical tests. IRT, in contrast, provides a flexible framework that is suited to a broad variety of testing scenarios.

Adaptive testing under IRT usually takes the following format:

1. Make an initial estimate for the participant’s ability. One approach is to use the population mean; another approach is to choose a purposefully low ability estimate so as to give test-takers an easy start to the test.
2. Repeat the following:

- Use the IRT model to choose one item from a repository of items termed an *item bank*. Items are preferred that deliver maximal information about the participant’s true ability level, subject to practical constraints (e.g. avoiding items already administered in the session).
- Administer the item.
- Re-estimate the participant’s ability using the IRT model.
- Terminate the test if and only if the stopping condition is satisfied (e.g. a certain number of items have been administered, or ability can be estimated with a certain precision).

Adaptive testing is a powerful tool for improving test efficiency, especially for tests intended to cater to a wide range of ability levels. Traditionally, if a fixed-item test is to perform well over a wide range of ability levels, it must contain many items spread over the corresponding difficulty range. As a result, any given test-taker must take many items that are poorly suited to their own ability level, reducing testing efficiency as a result. Adaptive testing addresses this problem by tailoring each test to the test-taker, ensuring that only maximally informative items are administered at each point. As a result, adaptive tests can be 50–80% shorter than corresponding non-adaptive tests while achieving matched reliability^19,38^.

Several existing musical listening tests use adaptive techniques, including the Adaptive Music Perception Test^39^, the Adaptive Melodic Discrimination Test^21^, the Adaptive Beat Alignment Test (A-BAT)^29^, and some sub-tests of the BAASTA^31^ and H-BAT^30^. However, IRT-based adaptive test implementations are rare, and none currently exist for beat perception. One reason for the slow adoption of IRT-based adaptive testing in musical as well as non-musical domains may be the expense traditionally involved in constructing such tests. IRT-based adaptive tests require large calibrated item banks, and traditionally this calibration requires each item to be administered to hundreds or thousands of participants, which can become very expensive.

### Automatic item generation

Automatic item generation can overcome this cost barrier to IRT-based adaptive testing by making test calibration much more efficient (see ref.^20^ for an overview). Instead of constructing and calibrating each item separately, an algorithmic procedure is defined for constructing items and estimating their psychometric parameters *a priori*. This makes test construction much more efficient, and allows item banks of effectively unlimited size, reducing the potential for exposure effects.

Automatic item generation depends on explanatory models of task performance linking psychometric parameters (in particular item difficulty) to observable properties of each item (*structural item features*). These explanatory models reflect the perceptual and cognitive processes that underlie task performance. Automatic item generation is therefore fundamentally a psychological endeavour: prior psychological knowledge can be leveraged to produce better generation paradigms, and the construction and validation of generation paradigms can conversely yield psychological knowledge that improves the test’s construct validity.

Music psychology stands to benefit from automatic item generation, but the technology is only recently being incorporated into musical tests^21^. Applying automatic item generation to beat perception testing has the exciting potential to improve both the efficiency and construct validity of beat perception testing.

## Test Design

### Generic item form

#### Paradigm

The CA-BAT uses a paradigm inspired by the perception component of the BAT^28^ and similar to that used in the Gold-MSI Beat Perception Test^6^ and the BAASTA^31^. The paradigm comprises a series of two-alternative forced-choice (2-AFC) trials. Each trial presents the participant with two versions of a musical track, both overlaid with a metronome-like beep track. In one version, the *target*, the beep track is exactly in time with the musical beat locations. In the other version, the *lure*, the beep track is displaced from the musical beat locations by a constant proportion of a beat. The participant’s task is to identify which extract is the target. This paradigm will be referred to as the *BAT paradigm*.

Previous tests mostly use a 1-AFC paradigm where participants categorise single extracts as ‘on-the-beat’ or ‘off-the-beat’. The 2-AFC paradigm is adopted here for two reasons. The first is that performance in 1-AFC paradigms depends heavily on two person parameters (discrimination and bias), and therefore is not modelled well by standard IRT approaches which only incorporate one person parameter (ability). Second, the 2-AFC response paradigm contributes to the robustness of test items in cases where the estimated canonical beat locations jitter somewhat around the true beat locations. The 1-AFC task is problematic in these scenarios because no single extract will ever be truly ‘on-the-beat’. The 2-AFC task performs better here, because ‘off-the-beat’ versions will have both jitter and systematic displacement, making them objectively more ‘off-the-beat’ than the ‘on-the-beat’ versions that they are being compared to.

#### Stimuli

Musical tracks were sourced from the ‘Audio Network’ production music library and chosen to represent a variety of musical genres and metres. Beat locations were estimated by recording experienced drummers tapping along to a selection of 45 musical tracks. Each drummer recorded at least three confident takes for each track. After correcting for equipment latency, canonical beat locations were established by averaging within each drummer the two recordings with the smallest difference in mean inter-tap interval and then averaging across drummers. Eight excerpts were rejected for low consistency within drummers (phase difference greater than 0.6 radians, i.e. 9.5% of a beat, within at least one drummer) or between drummers (phase difference greater than 0.7 radians, i.e. 11.1% of a beat, within any pair of drummers). A further five excerpts were rejected because the beat locations sounded inaccurate to the researchers. The resulting set numbered 32 musical tracks.

For the present study, each track was shortened to approximately five seconds in length, allowing some leeway to ensure that the extract was musically coherent, and overlaid with beep tracks. Each beep comprised a 20 ms sine tone with frequency 1000 Hz and a 10 ms fade-out. Initial piloting suggested that the perceptual salience (i.e. ease of hearing) of the beep track depended partly on the musical track it was mixed with, perhaps because of masking effects. Relative levels of beep tracks and musical tracks were therefore manually adjusted by one of the authors so as to equate beep-track perceptual salience across all musical tracks. These modifications were of the order 0–4 dB. The resulting mixes were then peak-amplitude normalised. In targets, the onset of each beep was aligned to the beat locations estimated from the drummers. In lures, each beep onset was temporally displaced a certain proportion (*P*) of the beat, either forwards or backwards, with $0 < P\le 0.5$. Each 2-AFC trial comprised one target and one lure, with the target either first or second, separated by two seconds of silence. Each resulting stimulus was approximately 12 s in length.

### Item-processing model

An explicit model of the perceptual and cognitive processes that underlie item processing is important both for construct validity and for effective automatic item generation^34,40^. Previous BAT studies do not present such a model, so one is suggested here.

Item processing begins with perceptual encoding, whereby the audio signal is translated from vibrations in air molecules into an internal representation of that audio signal. Through the process of auditory scene analysis^1^, this signal is split into two components, one belonging to the beep track and one belonging to the background musical track. The participant infers a metrical beat from the musical track by combining low-level auditory information (e.g. coinciding tone onsets from different instruments) with higher-level musical knowledge (e.g. knowledge that specific instruments tend to mark the musical beat), and represents the beep track relative to the resulting metrical grid. The participant then evaluates the extent to which beep onsets are aligned with the induced metrical grid. Applying this process to each stimulus version in the 2-AFC task produces two alignment estimates, one for each stimulus version. The final item response is arrived at by comparing these alignment estimates, with the participant selecting the stimulus version that achieves the best alignment. This process relies on working memory, especially for the first alignment estimate which needs to be retained until the second alignment estimate is complete.

The BAT paradigm is considered primarily to assess beat perception ability^28^. Worse beat perception means less reliable inferred beat locations, less reliable differences between target and lure, less reliable discrimination, and hence reduced test performance. However, abilities related to other stages of BAT processing could also affect performance. We try and minimise such effects: stimuli are played at comfortable volume to facilitate perceptual encoding, beep tracks are given salient pitch and loudness to facilitate auditory scene analysis, and delays between stimulus versions are kept short to facilitate working memory. Nonetheless, these subsidiary abilities could still affect performance, especially in the case of clinical impairment. It would be worth testing this possibility in future work.

### Relating item difficulty to item features

Effective automatic item generation relies on the ability to control item difficulty. A useful approach to this problem is to categorise structural item features into *radicals* and *incidentals*^41^. Radicals are features that affect difficulty, whereas incidentals are features that do not affect item difficulty. Manipulating radicals allows different item difficulties to be achieved, whereas manipulating incidentals introduces surface variation into the item bank, a useful tool for reducing exposure effects.

The item-processing model suggests potential radicals and incidentals for automatic item generation. The precise effects of these radicals and incidentals are quantified by calibrating the item response model. If the item response model delivers results consistent with the item-processing model, this supports the latter’s validity and hence the test’s construct validity.

#### Beep-track accuracy

The first hypothesised radical was beep-track accuracy (*d*_*r*_), the closeness with which the lure’s beep-track approximates the musical beat. Increasing the lure’s beep-track accuracy increases its similarity to the on-beat target, hence producing a harder discrimination task and increasing item difficulty. Because of the periodic nature of musical beat, the maximum beep-track displacement considered here is half a beat.

We originally intended to define beep-track accuracy as minus the amount of beep-track displacement, in units of beats. However, pilot experimentation suggested that the resulting radical did not satisfy the linear relationship with item difficulty required by the item response models used here. Instead, approximate linearity seemed to be achieved through the following heuristic transformation, adopted for the rest of the paper:

2 $${d}_{r}={cos}^{4}(\pi P)\,\,\,P:\,0 < P\le 0.5$$

where *P* is defined as *beep-track offset*, the amount of beep-track displacement in units of beats. This transformation reduces the effect of beep-track displacement on item difficulty for very low and very high displacement values. It would be worth investigating potential alternative transformations in future work.

#### Musical track

The second hypothesised radical was the musical track. Musical tracks might differ in the clarity of their beat locations; for example, some musical styles highlight beat locations with regular and prominent drum-beats, whereas other styles leave beat locations to be more implicit. These differences will affect the difficulty of the entrainment process. Musical tracks can also differ in terms of their underlying metre and tempo, and such differences are known to affect entrainment difficulty (e.g. ref.^42^).

An automatic item generation approach could try to disentangle the effects of different track features, such as tempo and metre, on item difficulty. This would be a difficult task, requiring a large number of musical tracks and a large amount of response data. Instead, it was decided to estimate the difficulty of each musical track separately, and treat the categorical variable *musical track* as a radical.

#### Displacement direction

One item feature as yet unexplored in BAT variants was the beep-track’s displacement direction, that is, whether the beep-track is displaced ahead of or behind the musical beat. Because of the lack of evidence to the contrary, it was hypothesised that displacement direction should be an incidental.

#### Stimulus order

There are two possible orders of stimulus presentation in the 2-AFC task: target-lure or lure-target. The order of stimuli within a 2-AFC task is rarely considered to be an important issue, and so it was hypothesised that stimulus order should be an incidental.

#### Other features

A number of other item features were kept constant so as to keep the psychometric model simple and minimise unexplained variation in item difficulty. These features included stimulus length, beep-track pitch and timbre, the perceptual salience of the beep-track, and the time between each stimulus version in the 2-AFC trial.

### Item response model

The relationship between item features and item parameters is characterised by an *explanatory item response model*^43^. This model is derived by imposing several constraints on the general IRT model described in equation (1). Most important from an automatic item generation perspective is the decomposition of the difficulty parameter (*c*_*j*_) into a linear predictor (${{\bf{c}}}^{{\rm{{\rm T}}}}{{\bf{x}}}^{(j)}\equiv {\sum }_{i}{c}_{i}{x}_{i}^{(j)}$). This linear predictor allows the difficulty of an item to be derived from its item features (*x*_*i*_).

Several additional constraints are imposed on the item parameters so as to improve the statistical efficiency of item parameter calibration.

1. The guessing parameter is constrained to the reciprocal of the number of response options, corresponding to the expected success rate if the participant were to answer randomly.
2. The inattention parameter is constrained to 1 on the understanding that inattention effects should be negligible for the short testing sessions employed in the present research.
3. The discrimination parameter is constrained to be equal for all items, a simplification commonly used in Rasch modelling^44^.

The resulting item response model takes the following form:

3 $$P({X}_{j}\mathrm{=1}|\theta ,{{\bf{x}}}^{(j)})=0.5+0.5\frac{\exp [a(\theta -{{\bf{c}}}^{{\rm{{\rm T}}}}{{\bf{x}}}^{(j)})]}{1+\exp [a(\theta -{{\bf{c}}}^{{\rm{{\rm T}}}}{{\bf{x}}}^{(j)})]}$$

where *X*_*j*_ denotes the scored response of the test-taker to item *j* (1 = correct, 0 = incorrect), *θ* is the test-taker’s ability parameter, ${{\bf{x}}}^{(j)}$ is the vector of item features for item *j* (including an intercept, and any categorical features dummy-coded), **c** is a vector of regression coefficients, and *a* is the discrimination parameter.

Calibration of this item response model involves estimation of the discrimination parameter (*a*) and the feature effects (**c**). Two estimation methods are used in the present research: a linear regression approach and a generalised linear mixed model (GLMM) approach.

The linear regression approach is a two-stage process. In the first stage, a traditional IRT model (e.g. equation (1)) is fit to response data, and separate item difficulties are estimated for every item tested. In our case the IRT model is a version of equation (1) where the inattention parameter (*d*_*j*_) is constrained to 1 and the discrimination parameter (*a*_*j*_) is constrained to be shared by all items. This model is sometimes termed a constrained three-parameter logistic (3-PL) model^45^. A linear regression is then conducted to predict item difficulty from item features; the resulting regression coefficients can then be used as the parameters *c*_*i*_ in equation (3). This approach has been used before by ref.^46^. An advantage of this approach is that it enables traditional IRT model diagnostics, which are useful tools for developing and validating IRT models. Disadvantages include the difficulty of model calibration when participants take different item sets, and the fact that variable imprecision in item parameter estimates is not propagated through the regression model.

The GLMM approach is based on the observation that the desired item response model (equation (3)) can be specified as a GLMM if the population distribution of test-taker ability (*θ*) is modelled as a Gaussian. The relationship between GLMMs and IRT is discussed in greater detail in ref.^47^. We will just make a couple of additional observations:

1. Item response models with non-zero guessing parameters or inattention parameters can be specified as GLMMs through appropriate modification of the link function, as long as the guessing, inattention, and discrimination parameters are constrained to be invariant between items.
2. When converting parameter estimates from the GLMM metric to the traditional IRT metric ($\theta \sim {N}(0,1)$), the IRT discrimination parameter (*a*) is equal to the standard deviation of the GLMM test-taker random intercept, and the item effects (*c*_*i*_) are equal to the corresponding GLMM effects multiplied by −1/*a*.

The GLMM approach is much more flexible for calibration, in that participants can take arbitrary item sets. One limitation, however, is the unavailability of many traditional IRT model diagnostics.

We developed our item response model over the course of two studies, described below. The first study was exploratory, and used the linear regression approach to test the main radical, beep-track accuracy. The second study explored a wider range of radicals and incidentals using the GLMM approach. Data and example code for these item response models are available at 10.5281/zenodo.1211116.

## Studies

### Study 1: Exploratory study

This study represented an initial investigation into the possibility of manipulating BAT item difficulty. For simplicity, only one item feature was modelled explicitly: beep-track accuracy. The aim of the study was to investigate the suitability of the BAT paradigm for IRT modelling, and to see how well item difficulty could be predicted from beep-track accuracy.

### Method

#### Participants

The participant group comprised 197 self-recruited individuals (87 female) between the ages of 18 and 75 (*M* = 26.2, *SD* = 9.3). The majority of participants were students. Participants were recruited by word-of-mouth and via social media, and participation was rewarded by entry into a prize draw for a £100 gift voucher as well as the promise to acquire one’s own ‘Beat Perception Score’.

#### Materials

The test comprised 32 items in total, produced by randomly combining the original 32 musical tracks with 32 equally spaced levels of beep-track accuracy (*d*_*r*_), with $0.25\le {d}_{r} < 1$ (piloting suggested that items with *d*_*r *_< 0.25 were too easy to be worth testing). Sixteen randomly selected items had their beep track displaced ahead, the other 16 behind; likewise, 16 randomly selected items had the target come first, while the other 16 had the target come second. Otherwise, all items followed the generic item form described above.

#### Procedure

The experiment took place online over a two-week period, with each participant agreeing to wear headphones and to take the test in a quiet room free from interruptions. The entire testing session was run within the online platform Concerto^48^, beginning with the BAT items and concluding with a short series of questionnaires; on average, sessions lasted approximately 10 minutes. Before the testing session, participants were played some example music and instructed to adjust the volume of their headphones to a comfortable level. BAT testing began with a training phase, including instructions, audio demonstrations, and two practice questions, before proceeding to the data collection phase. In the data collection phase, participants were administered the 32 test items in randomised order, without item-to-item feedback. After completing the BAT items, participants took the 7-item Musical Training component of the Gold-MSI self-report questionnaire^6^, alongside a short questionnaire concerning their age, gender, and occupational status. Upon completion participants were presented with their total BAT score.

#### Ethics

All experimental protocols in this and subsequent studies were approved by the Ethics Committee of Goldsmiths, University of London, and all experiments were performed in accordance with the relevant guidelines and regulations. Informed consent was obtained from all participants prior to participation.

### Results

**Figure 2:**
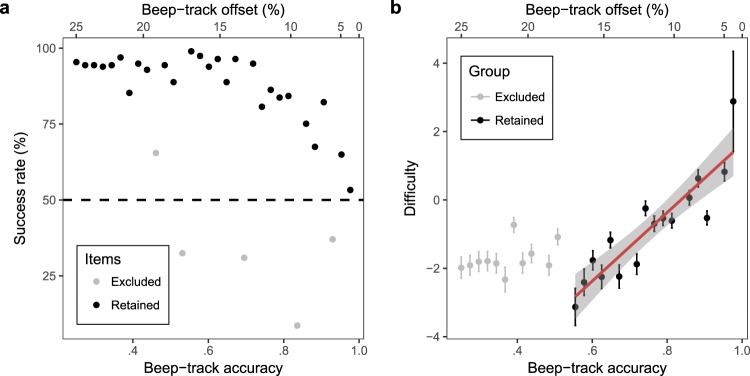
Item-wise statistics for Study 1. Item difficulty generally increases with beep-track accuracy, excepting several outliers and a floor effect for accuracies below 0.55. (**a**) Success rate (averaged over all participants) as a function of beep-track accuracy or beep-track offset. Chance success rate (50%) is marked by the dashed line. The five excluded tracks are marked grey. (**b**) Difficulty as a function of beep-track accuracy and beep-track offset, having removed the five excluded tracks. Items with beep-track accuracies less than 0.55 are marked grey. Error bars denote one standard error. The fitted regression line is plotted with the standard error of its predictions shaded in grey.

Prior to any IRT analysis being conducted, the data were inspected to investigate item-wise success rate (averaged over all participants) as a function of the accuracy of the manipulated beep track (Fig. 2a). These initial results suggested a negative association between success rate and beep-track accuracy, as hypothesised. However, several items displayed outlying success rates, with four items scoring below chance (50%), and one scoring above-chance but still substantially worse than its neighbours (Fig. 2a, grey items). These outliers seemed likely to be due to inaccurate beat location data or misleading features in the underlying tracks, and so these five musical tracks were removed from subsequent analyses and excluded from the future CA-BAT.

Having excluded the five outliers, an IRT model was fit to the remaining data. This model took the form of a 3PL model with guessing parameter constrained to 1/2, fit with approximate marginal maximum likelihood using the ltm package^49^ in the statistical software environment R^50^.

The validity of the resulting IRT model was tested in several ways. Model fit was assessed using Yen’s^51^ Q1 statistic with 10 ability groups, estimating the test statistic’s distribution under the null hypothesis with 500 Monte Carlo samples, and calculating significance levels using Bonferroni correction. No items exhibited statistically significant levels of poor fit ($p > 0.05$). Model fit and conditional independence were then assessed by computing model fit on the two- and three-way margins^52^. This procedure involves looking at all pairs and triplets of items and testing whether their combined success rate differs significantly from that predicted by the IRT model. 1404 pairs of items and response patterns were examined in total, out of which only 7 pairs (0.50% of the total) were flagged for poor fit by Bartholomew’s^52^ criterion. Similarly, only 530 out of 23400 (2.26%) of triplets were flagged for the three-way margins. These results suggested that the items exhibited good fit and conditional independence. Lastly, the assumption of unidimensionality was tested using modified parallel analysis^53^. This procedure involved calculating the tetrachoric correlation matrix for the response data, deriving its second eigenvalue, and comparing the eigenvalue to its simulated distribution under the null hypothesis, using a Monte Carlo simulation with 500 samples. The analysis found no evidence for multidimensionality (*p* = 0.822).

Participant abilities were estimated from the IRT model using expected a posteriori estimation and compared with their Gold-MSI Musical Training scores. BAT scores and Musical Training scores exhibited a moderate positive correlation ($r(195)=0.454,\,\,p < 0.001$, 95% CI = [0.335, 0.558]).

Explanatory item response model parameters were then estimated on the basis of the IRT model using the linear regression approach. The discrimination parameter was taken directly from the IRT model, and had a value of 1.616 (*SE* = 0.158). Visual inspection of the relationship between beep-track accuracy and item difficulty suggested that the trend was linear, but that difficulty reached a lower asymptote at a beep-track accuracy of about 0.55. Beep-track accuracies lower than 0.55 were therefore excluded from the explanatory model. Coefficients for item effects (*c*_*i*_) were then estimated for the explanatory item response model (equation (3)) by conducting a linear regression to predict item difficulty from beep-track accuracy (Fig. 2b, Table 1). The resulting model had good fit (Adj. *R*^2^ = 0.80, *F*(1,14) = 61.11, $p < 0.001$). Beep-track accuracy significantly predicted item difficulty, with higher beep-track accuracies producing higher difficulty, as hypothesised (*β* = 10.025, *t*(14) = −7.817, $p < 0.001$).

**Table 1:** Item response model parameter estimates (Study 1).

*i*	${{\boldsymbol{x}}}_{{\bf{i}}}^{({\bf{j}})}$	*B*_*i*_	*SE*(*B*_*i*_)	*β*_*i*_	*SE*(*β*_*i*_)	*c*_*i*_	*SE*(*c*_*i*_)	*p*
0	Intercept	−8.388	0.983	*NA*	*NA*	−8.388	0.983	< 0.001
1	Beep-track accuracy	10.025	1.282	0.902	0.115	10.025	1.282	< 0.001

*Notes*. *i* is a generic index. ${{\rm{x}}}_{i}^{(j)}$ denotes the *i*th element of vector **x**^(*j*)^ (equation (3)), and corresponds to the *i*th item feature. *B*_*i*_ denotes an unstandardised regression coefficient. *SE*(*B*_*i*_) denotes the standard error associated with the estimation of *B*_*i*_, and analogous definitions hold for *SE*(*β*_*i*_) and *SE*(*c*_*i*_). *β*_*i*_ denotes a fully standardised regression coefficient. c_*i*_ denotes the *i*th element of vector **c** (equation 3), and identifies the contribution that item feature ${x}_{i}^{(j)}$ makes to item difficulty.

### Discussion

The purpose of this study was to investigate the suitability of the BAT paradigm for IRT modelling, and to see how well item difficulty could be predicted from beep-track accuracy. A variety of standard IRT tests showed that the BAT items satisfied the key assumptions of IRT, once a number of outliers had been removed. Moreover, the results showed that the difficulty of BAT items might be predicted fairly well by a linear function of beep-track accuracy, as long as a sufficiently constrained range of beep-track accuracies was used and a number of outlying musical tracks excluded. Nonetheless, 20% of the variance in item difficulty remained unexplained, suggesting the influence of additional item features beside beep-track accuracy. Moreover, given that 16 items had been manually excluded (five for outlying success rates, the rest for too-low beep-track accuracies), the model could only be treated as exploratory.

This study also provided evidence for the construct validity of the CA-BAT paradigm. The moderate correlation of ability scores with musical training supports the conception of beat perception as fundamental musical skill, and is consistent with previous studies of beat perception ability (e.g. ref.^6^). Moreover, the observed relationship between item difficulty and beep-track accuracy is consistent with the hypothesised item-processing model for the CA-BAT, supporting the test’s construct representation and construct validity^34^.

### Study 2: Calibrating the CA-BAT

The purpose of this study was to develop an improved psychometric model for the CA-BAT. This involved making several changes to the previous design. First, a new item bank was generated that explored more combinations of item features than were present in Study 1. Second, the range of beep-track accuracies was constrained to the more appropriate range identified in Study 1. Third, item response model parameters were estimated with the GLMM approach instead of the linear regression approach. The latter approach requires many responses for each combination of features, which would have been impractical here.

### Method

#### Participants

The participant group was drawn from two samples: one large group who took the test online (*N* = 223) and one small group of schoolchildren (*N* = 6; unfortunately participation numbers were lower than expected for this group). Ages and genders were not collected for the schoolchildren, but in the online group ages ranged from 18 to 68 (*M* = 32.8, *SD* = 12.8), with 52.8% self-reporting as female. Online participants were recruited in a similar manner to Study 1.

#### Materials

The item bank contained 2,916 items in total, produced by factorially combining four variables:

- 27 musical tracks (five from the original 32 had been discarded in Study 1);
- 27 equally spaced levels of beep-track accuracy (*d*_*r*_), with $0.5\le {d}_{r}\mathrm{ < 1}$;
- displacement direction (two levels: ahead or behind);
- stimulus order (two levels: target-lure or lure-target).

Otherwise, all items followed the generic item form described previously.

#### Procedure

Data collection took place over a one-month period using the Concerto platform^48^. Online participants agreed to wear headphones and to take the test in a quiet room free from interruptions; the schoolchildren took the test in a quiet classroom with headphones. All participants were instructed to adjust the volume of their headphones to a comfortable level prior to the testing session. For the online participants, the overall session structure was identical to that of Study 1; in contrast, the schoolchildren took the test as part of a c. 45-minute battery of other tests and questionnaires, the results of which are not reported here. After completing the training phase, all participants answered 27 items, which were randomly selected from the item bank under the following constraints: a) each participant heard each musical track exactly once; b) each participant heard each level of beep-track accuracy exactly once.

### Results

**Figure 3:**
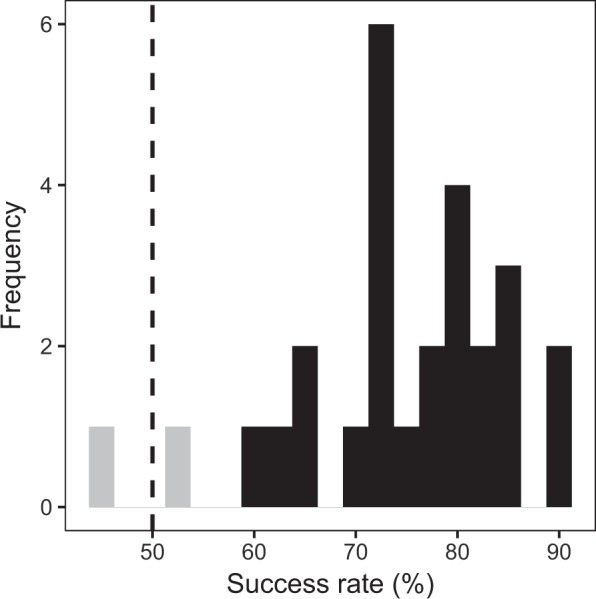
Distribution of track-wise success rates (Study 2). Chance success rate (50%) is marked by the dashed line. The two excluded songs are marked grey.

Study 1 had identified a number of items with unexpectedly low success rates. The data from Study 2 were screened for similar cases by aggregating success rates for each musical track (Fig. 3). On the whole track-wise success rates appeared approximately normally distributed about a mean of 76%, but two musical tracks scored approximately at or below chance (52% and 46% respectively). These two tracks were therefore removed from the analysis as well as from the future CA-BAT.

The item response model in equation (3) was then fit to the remaining data using the GLMM approach. Estimation was carried out using the R^50^ packages lme4^47^ and psyphy^54^.

The item response model was constructed incrementally by adding radicals one at a time, allowing the assessment of the individual contributions of each radical. Four model versions were planned, each adding a new potential radical in the order of their hypothesised importance (Table 2). Sequential comparisons with likelihood-ratio tests would then provide a statistical measure of the significance of each radical’s contribution.

**Table 2:** Planned model specifications (Study 2).

Model	Fixed effects	Random effects
A	beep-track accuracy	participant (intercept) item (intercept)
B	same as A + musical track	same as A
C	same as B + displacement direction	same as A
D	same as C + stimulus order	same as A

In practice, model convergence issues necessitated some revision of the planned model specifications. Model A failed to converge, prompting the removal of the item-wise random intercept from all models. Model B also failed to converge, prompting the revision of musical track from a fixed effect to a random slope with beep-track accuracy, including an intercept-slope correlation parameter. Model C had no convergence problems, but Model D failed to converge.

The revised models were then compared with likelihood-ratio tests (Table 3). Model C significantly outperformed Model B, which significantly outperformed Model A, supporting the predictive importance of both musical track and displacement direction. Because Model D did not converge, the influence of stimulus order was instead examined with a chi-squared test at the response level that tested for an association between stimulus order and correctness of response. As hypothesised, stimulus order did not have a significant effect on success rate (*χ*^2^(1) = 0.373, *p* = 0.541). These results indicated that the best-performing model was Model C, and so Model C was adopted as the final item response model.

**Table 3:** Fitted model specifications (Study 2).

Model	Fixed effects	Random effects	AIC	BIC	LR test
A	beep-track accuracy	participant (intercept)	5877.3	5897.2	*NA*
B	same as A	A + track (slope with accuracy)	5657.7	5697.7	*χ*^2^(3) = 225.51, $p < 0.001$
C	B + displacement direction	same as B	5633.1	5679.6	${\chi }^{2}\mathrm{(1)}=\mathrm{26.67,}\,p < 0.001$

*Note*. Likelihood-ratio (LR) tests are each carried with respect to the model in the immediately preceding row, and characterise the extent to which the new model better accounts for the data than the previous model.

Parameter estimates for the final model are given in Tables 4 and 5. The discrimination parameter (*a*) is set to the standard deviation of the participant intercept (Tables 5, 1.166), and GLMM fixed effects (*B*_*i*_) are converted to the metric of the desired item response model (*c*_*i*_) by dividing by −*a* (Table 4). Consistent with Study 1, the results indicate that increased beep-track accuracy yields increased difficulty. In addition, the results indicate that item difficulty increases when the beep-track is displaced ahead of the beat rather than behind the beat.

**Table 4:** Final model (Model C) fixed-effect parameters.

*i*	${{\boldsymbol{x}}}_{{\bf{i}}}^{({\bf{j}})}$	*B*_*i*_	*SE*(*B*_*i*_)	*β*_*i*_	*SE*(*β*_*i*_)	*c*_*i*_	*SE*(*c*_*i*_)
0	Intercept	6.287	0.585	*NA*	*NA*	−5.393	0.502
1	Beep-track accuracy	−8.448	0.611	−1.217	0.088	7.247	0.524
2	Displacement direction	0.671	0.130	*NA*	*NA*	−0.576	0.112

*Notes. i* is a generic index. ${{\rm{x}}}_{i}^{(j)}$ denotes the *i*th element of vector **x**^(*j*)^ (equation (3)), and corresponds to the *i*th item feature. *B*_*i*_ denotes the unstandardised regression coefficient for the corresponding fixed effect in the mixed-effects logistic regression model. *SE*(*B*_*i*_) denotes the standard error associated with the estimation of *B*_*i*_, and analogous definitions hold for *SE(β*_*i*_) and *SE*(*c*_*i*_). *β*_*i*_ denotes a partially standardised logistic regression coefficient, where standardisation is achieved by multiplying the unstandardised coefficient by the standard deviation of the predictor variable. *c*_*i*_ denotes the *i*th element of vector **c** (equation (3)), and identifies the contribution that item feature ${x}_{i}^{(j)}$ makes to item difficulty. ${x}_{2}^{(j)}$ (displacement direction) is coded with 1 denoting ‘behind-the-beat’ and 0 denoting ‘before-the-beat’.

**Table 5:** Final model (Model C) random effects.

Parameter	Estimate
Participant (intercept)	1.166
Track (intercept)	2.121
Track (slope with accuracy)	1.477
Track (intercept-slope correlation)	−1.000

Note. No standardisation is applied to the random-effect parameters.

The estimated random-effect coefficients are fairly large (Table 5), implying that the choice of musical track significantly affects item difficulty. However, the perfect negative correlation between the intercept and slope coefficients for musical track somewhat mitigates the relationship between musical track and item difficulty. This large negative correlation is necessitated by the fact that all musical tracks must asymptotically approach the same ‘impossible’ difficulty level as beep-track accuracy increases to 1. This constraint reduces one degree of freedom from track-wise variation, and results in only one effective random parameter for each track.

Theoretically both fixed effects and random effects can be incorporated into the linear predictor (**c**^Τ^**x**^(*j*)^). The potential advantage would be increased model expressivity, allowing the model to generate different difficulty predictions for different musical tracks. The potential disadvantage would be overfitting, a result of the dataset being too small for accurate parameter estimation. Pilot testing suggested that including the random effects reduced test reliability, and so it was decided to omit these effects. The resulting item response model estimates the difficulty of each item solely on the basis of its beep-track accuracy and the direction of the beep-track’s displacement:

4 $${\rm{d}}{\rm{i}}{\rm{f}}{\rm{f}}{\rm{i}}{\rm{c}}{\rm{u}}{\rm{l}}{\rm{t}}{\rm{y}}={{\bf{c}}}^{{\rm{T}}}{\bf{x}}=-5.393+(7.247\times {\rm{a}}{\rm{c}}{\rm{c}}{\rm{u}}{\rm{r}}{\rm{a}}{\rm{c}}{\rm{y}})+(-0.576\times {\rm{d}}{\rm{i}}{\rm{r}}{\rm{e}}{\rm{c}}{\rm{t}}{\rm{i}}{\rm{o}}{\rm{n}})$$

where ‘direction’ is coded with 1 for ‘behind the beat’ and 0 for ‘ahead of the beat’.

### Discussion

The purpose of this study was to improve the psychometric model for the CA-BAT. The results indicate that incorporating additional item features resulted in a better model, with both musical track and displacement direction providing statistically significant improvements in model fit. The resulting model can now be used to generate item parameter estimates for the CA-BAT item bank.

Increased beep-track accuracy produced more difficult items, as hypothesised and consistent with the original item-processing model. Likewise, different musical tracks produced different item difficulties, as predicted from the item-processing model.

The observed effect of displacement direction–that item difficulty increases when displacement direction is ahead of the beat–was unexpected. The result implies that beeps sound more “in time” when they are ahead of the beat, rather than behind the beat. This makes an interesting parallel with the phenomenon of negative mean asynchrony found in sensorimotor synchronisation studies, where it is established that individuals (especially musically untrained ones) have a general tendency to tap slightly ahead of the beat (e.g. ref.^55^). However, the observed effect could also stem from systematic biases in the canonical beat locations.

These relationships between item features and item difficulty provide useful information concerning the construct validity of the CA-BAT. To the extent that these relationships are consistent with the proposed item-processing model, it can be claimed that the CA-BAT paradigm is well-understood, and has good *construct representation*^34^. The results presented here suggest fairly good construct representation for the CA-BAT, as both the effects of beep-track accuracy and musical track are consistent with the proposed item-processing model. However, the effect of displacement direction is not yet fully understood, and deserves further exploration.

This study showed that musical track significantly affects item difficulty, and so ideally musical track should be explicitly included in item difficulty predictions. Unfortunately it proved difficult to estimate the necessary fixed-effect parameters to achieve this. Future work could address this problem in several ways. One approach would be to compile a larger set of response data; perhaps more data would produce greater stability in the parameter estimates. A second approach would be to use penalised regression, which again should induce greater parameter stability. In the meanwhile, including musical track as a random effect improves overall model fit and therefore should improve the remaining parameter estimations.

### Study 3: Laboratory validation of the CA-BAT

Study 3 was intended to investigate the behaviour of the CA-BAT in a typical laboratory setting. One aim was to investigate the test’s reliability, that is, the extent to which it delivers consistent ability estimates. An emphasis was placed on investigating reliability as a function of test length, to see whether the test could perform well when shortened.

A second aim was to investigate the test’s *nomothetic span*, the way in which CA-BAT scores relate to different psychometric measures. Nomothetic span is an important aspect of construct validity^34^, the question of what latent trait(s) the test measures. The comparison measures used in this study all derive from the self-report component of the Gold-MSI^6^. This tool assesses a multi-faceted conception of musical sophistication comprising five sub-components, *Active Engagement*, *Emotions*, *Musical Training*, *Perceptual Abilities*, and *Singing Abilities*, as well as one general component, termed *General Sophistication*. A similar comparison had been performed in a previous study for the Gold-MSI Beat Perception Test^6^, finding small positive correlations for each component (Active Engagement, *r* = 0.224; Emotions, *r* = 0.218; Musical Training, *r* = 0.356; Perceptual Abilities, *r* = 0.342, Singing Abilities, *r* = 0.305). The present study provided an opportunity to attempt to replicate these results.

### Method

#### Participants

The participant group numbered 71 psychology undergraduates who participated for course credit. Ages ranged from 18 to 40 years (*M* = 22.73, *SD* = 4.61). Forty-five self-reported as female, 25 as male, and one declined to report a gender.

#### CA-BAT

The item bank for the CA-BAT was constructed by extending the item bank of Study 2 to cover 100 levels of beep-track accuracy (*d*_*r*_), with $0.5\le {d}_{r} < 1$. Point estimates were made for all item parameters according to the results of Study 2. The resulting item bank numbered 10,000 items.

The CA-BAT was implemented within the Concerto platform^48^, an extension to the statistical software environment R^50^ for computerised psychometric testing, and making use of the R package catR, which provides a set of algorithms for adaptive test administration^35^.

The first item administered was constrained to be the same for all participants (aside from stimulus order, which was allowed to vary); this item was chosen to correspond to an average ability level, with a difficulty of −0.01. After each response, the participant’s ability would be re-estimated using Bayes modal estimation with a Gaussian prior of mean 0 and standard deviation 1. The next item would then be randomly selected from the subset of items in the item bank being maximally close in difficulty to the current ability estimate (Urry’s criterion^35^), with the constraint that the same musical track could not be heard twice. The test would terminate after 25 items, at which point the ability estimate would be recomputed using weighted maximum-likelihood estimation^56^, thereby removing the bias of the Bayesian prior.

#### Procedure

The experiment comprised two waves termed Wave 1 (test session) and Wave 2 (re-test session). All individuals that participated in Wave 1 were invited to participate in Wave 2 between 7 and 14 days later. Nineteen individuals declined this opportunity, leaving 52 individuals to participate in both waves.

The Wave 1 session comprised the new CA-BAT, the Gold-MSI self-report inventory, and an unrelated tuning perception experiment. The entire session lasted approximately 25 minutes. The Wave 2 session only comprised the CA-BAT and lasted no more than 15 minutes.

Subsequent to online data collection, a simulation experiment was conducted to assess the consistency of the IRT model with real-world test performance. This simulation was conducted using the R package catR^35^, with a virtual participant group constructed by taking each ability estimate from Wave 1 of empirical data collection and creating 250 participants with that same ability. This virtual participant group took the test twice to allow the assessment of test-retest reliability.

### Results

#### Reliability

**Figure 4:**
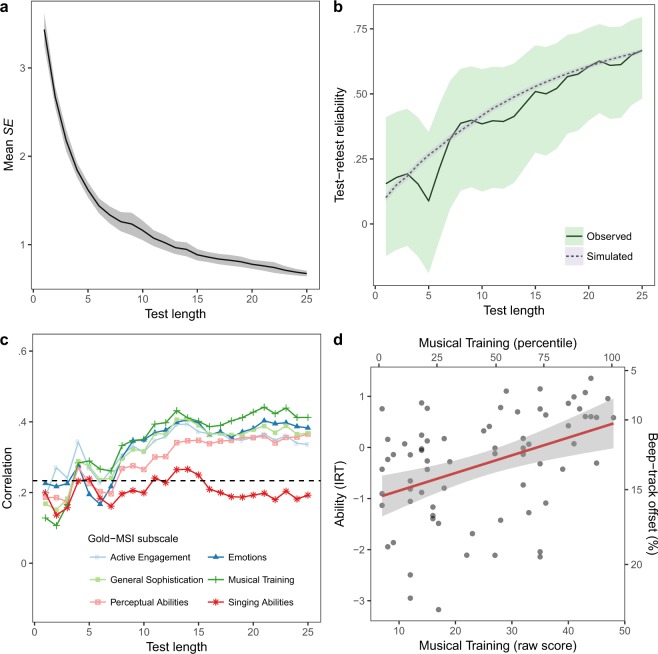
Study 3 (laboratory testing) results. The test displays good reliability (mean *SE* and test-retest reliability) and nomothetic span (correlations with related measures). (**a**) Mean standard error (*SE*) of CA-BAT ability estimates plotted as a function of test length. The shaded ribbon marks the 95% confidence region. (**b**) Test-retest reliability (Pearson correlation) plotted as a function of test length. The shaded ribbons mark 95% confidence regions. (**c**) Correlations between CA-BAT ability estimates (Wave 1) and sub-scales of the Gold-MSI self-report questionnaire, plotted as a function of test length. The dotted line marks the threshold for two-tailed statistical significance at a level of $p < 0.05$. (**d**) CA-BAT performance (Wave 1, after 25 items) as a function of Musical Training scores from the Gold-MSI self-report questionnaire. A least-squares regression line is plotted with the standard error of its predictions shaded in grey. Musical Training raw scores are mapped to population percentiles reported in the original Gold-MSI study^6^. CA-BAT performance is represented both as ability estimates and as corresponding beep-track offsets for ‘ahead-of-the-beat’ items.

IRT provides a mechanism for estimating the imprecision of its own ability estimates. Figure 4a presents this information, plotting the mean estimated standard error (*SE*) of ability estimates as a function of test length (shorter test lengths than 25 items were simulated by making a weighted maximum-likelihood ability estimate after the appropriate number of items). Mean *SE* started high but quickly decreased as test length increased, reaching 1.62 [1.54, 1.70] after 5 items, 0.88 [0.82, 0.95] after 15 items, and 0.67 [0.64, 0.70] after 25 items (95% confidence intervals in square brackets).

Reliability was also estimated empirically by calculating test-retest correlations. Pearson correlations between CA-BAT scores from Waves 1 and 2 are plotted in Fig. 4b, for both observed and simulated participants. Observed test-retest reliability started low but increased gradually as test length increased, with *r*(50) = 0.09 [−0.19, 0.35] after 5 items, *r*(50) = 0.51 [0.27, 0.69] after 15 items, and *r*(50) = 0.67 [0.48, 0.80] after 25 items. Simulated test-retest reliabilities showed a similar pattern, staying well within the 95% confidence intervals of the observed reliabilities.

#### Nomothetic span

Evidence for nomothetic span comes from Pearson correlations between scores on the CA-BAT and on the six Gold-MSI sub-scales (Fig. 4c). All reported correlations use CA-BAT scores from Wave 1. Consistent with ref.^6^, CA-BAT scores correlated positively with all Gold-MSI sub-scales, except in the case of Singing Abilities where the correlation was not statistically significant, even after 25 CA-BAT items (*r*(69) = 0.19, *p* = 0.11, 95% CI = [−0.04, 0.41]). Correlations with the other five sub-scales began below the margin of significance ($p > 0.05$), but gradually rose as test length increased, with significant correlations for all five after about 8 CA-BAT items. Soon after this point the correlations seem to have plateaued at around *r* = 0.4. As with ref.^6^, the highest correlation was with Musical Training (after 25 items $r(69)=0.41,p < 0.001,95{\rm{ \% }}\,{\rm{C}}{\rm{I}}=[0.20,\,0.59]$).

The observed relationship between Musical Training and CA-BAT scores is plotted in Fig. 4d. Because participant ability and item difficulty share a common metric and because difficulty is predicted from item features, it is possible to associate participant and item characteristics directly. In this respect, Fig. 4d shows that people with low musical training scores (<10) are best matched to a beep-track offset of about 15%, while participants with high musical training scores (>45) are best matched to a smaller offset of about 10%. Figure 4d also suggests that CA-BAT abilities are more variable for lower levels of musical training than for higher levels of musical training.

### Discussion

These results provide evidence that the 25-item CA-BAT performs well as a musical ability test: it displays acceptable levels of reliability, and the fact that CA-BAT scores positively correlate with other measures of musical sophistication is evidence for the test’s construct validity. Moreover, the consistency of observed and simulated reliability estimates supports the validity of the underlying psychometric model.

It is difficult to make a definite statement about the reliability of the test compared to previous BAT implementations, since most studies presenting BAT variants have not tested reliability. One exception is ref.^6^, which reports a test-retest reliability of 0.63 with 34 participants for the Gold-MSI Beat Perception Test. The 25-item CA-BAT performs slightly better (*r* = 0.67), but this comparison means little since the two studies used different sample groups and different reliability measures. Nonetheless, the evidence suggests that the 25-item CA-BAT achieves state-of-the-art performance. Further improvements to reliability might be achieved by increasing test length past 25 items. However, this would involve repeating musical tracks within participants, which might introduce unwanted familiarity effects.

Figure 4b provides useful information concerning the potential for shortening the CA-BAT. There is no clear plateau in reliability within the range of test lengths examined, meaning that there is no obvious length to which the test might be shortened. Instead, there seems to be a continuous trade-off between test length and reliability. The most practical test length is likely to vary between use cases; Fig. 4b provides a way for that decision to be made on a principled basis.

The low correlation between CA-BAT scores and singing abilities is theoretically plausible: singing is a performance mode where note onsets are relatively poorly defined and precise temporal synchronisation is often unimportant. However, it should be noted that a significant correlation (*r* = 0.305) was found between Beat Perception and Singing Abilities scores in ref.^6^. Perhaps the lack of correlation observed in the present study is an artefact of the limited sample size.

### Study 4: Online validation of the CA-BAT

Increasing numbers of psychological studies use online participant testing for its time efficiency, scalability, and low cost. The primary aim of Study 4 was to investigate the suitability of the CA-BAT for such testing scenarios. A secondary aim of the study was to investigate further the nomothetic span of the CA-BAT, comparing CA-BAT scores with scores from two other listening ability tests: a melodic discrimination test and a temporal order discrimination test.

Melodic discrimination (the ability to discriminate between similar versions of a melody) is a core musical skill that is often assessed in musical aptitude and ability tests^21,57^. Moderate correlations between CA-BAT scores and melodic discrimination scores were predicted, consistent with ref.^6^.

Temporal order discrimination tests are low-level psychoacoustic tests that assess the participant’s ability to discriminate the temporal order of two auditory sounds played in quick succession. This paradigm is relevant to the CA-BAT in that it replicates a key part of the BAT paradigm (comparing the relative temporal position of two almost-synchronised auditory objects) while removing the cognitive task of auditory entrainment. Investigating CA-BAT correlations with temporal order discrimination scores therefore provides a way of investigating which of these cognitive processes drives individual differences in the CA-BAT. A high correlation in test scores would imply that differences in CA-BAT scores are driven by differences in order-comparison ability, whereas a low correlation would imply that CA-BAT scores are driven by entrainment ability.

### Method

#### Participants

A sample group of 185 participants was recruited by the market research company ‘Qualtrics’ so as to be broadly representative of the UK in terms of age (*M* = 43.1, *SD* = 13.9), gender (93 female, 92 male), occupation, and geographic location. No participants reported hearing problems.

#### CA-BAT

The CA-BAT took an identical form to Study 3.

#### Melodic discrimination test

This study used the adaptive melodic discrimination test presented in ref.^21^. This adaptive test uses a three-alternative forced-choice (3-AFC) paradigm where the participant has to determine which of three melody versions differs from the others. A test length of 20 was used along with Bayes modal ability estimation.

#### Temporal order discrimination test

The temporal order discrimination test was modelled after the Temporal Order for Tones test of ref.^7^. This test uses a 3-AFC procedure, where the task is to discriminate the order of two equal-duration ‘target’ tones, one that is 550 Hz in frequency and one that is 710 Hz in frequency. These tones are played without a gap between them, and are preceded and followed by 625 Hz ‘leader’ and ‘trailer’ tones, each 100 ms in duration. The original test is non-adaptive, but the present study used an adaptive version based on an IRT formulation of the adaptive maximum-likelihood procedure^37^, estimating item difficulty as a function of the duration of the target tones, using Bayes modal ability estimation, and selecting successive items using Urry’s criterion^35^. The test was implemented using R^50^, catR^35^, and Concerto^48^. An identical implementation was used by ref.^21^.

#### Procedure

Participation took place in two waves termed Wave 1 (test session) and Wave 2 (re-test session). All individuals that participated in Wave 1 were invited to participate in Wave 2 approximately one week later, with 42 participation slots available on a first-come first-served basis.

Wave 1 began with the adaptive melodic discrimination test, continued with the CA-BAT, and concluded with a questionnaire comprising the Musical Training sub-scale of the Gold-MSI as well as some demographic questions (age, gender, occupation). This wave lasted approximately 30 minutes in total.

Wave 2 took the same format except that the questionnaire was replaced with the temporal order discrimination test. The wave lasted about 20 minutes in total.

This online experiment was replicated by a simulation experiment in the same manner as Study 3. As before, a virtual participant group was constructed by up-sampling ability estimates from Wave 1 by a factor of 250. Two 25-item test sessions were then simulated for these virtual participants.

### Results

#### Reliability

**Figure 5:**
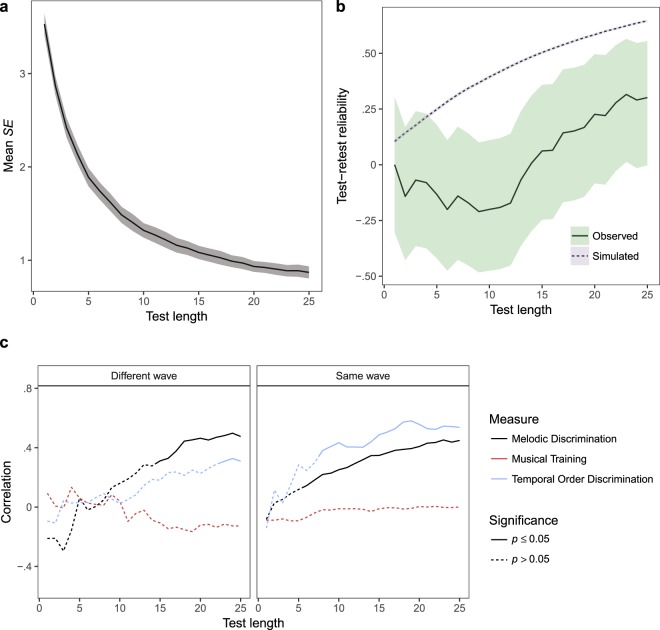
Study 4 (online testing) results. Test reliability (mean *SE* and test-retest reliability) is surprisingly low. Nomothetic span (correlations with related measures) is unexpectedly sensitive to testing session. (**a**) Mean standard error (*SE*) of ability estimates as a function of test length. The shaded ribbon marks 95% confidence intervals. (**b**) Test-retest reliability (Pearson correlation) as a function of test length. The shaded ribbons mark 95% confidence regions. (**c**) Correlations between CA-BAT scores and other measures as a function of test length and split according to whether the measures were administered in the same or different waves. Significance values are two-tailed and not adjusted for multiple comparisons.

Figure 5a plots the mean estimated standard error (*SE*) of ability estimates as a function of test length, as estimated by the IRT model. The profile is largely similar to that observed in Study 3 (Fig. 4a), but with *SE* values increased by about 20–25%. Mean *SE* started high but quickly decreased as test length increased, reaching 1.89 [1.79, 1.99] after 5 items, 1.08 [1.01, 1.15] after 15 items, and 0.87 [0.81, 0.93] after 25 items (95% confidence intervals in square brackets).

Test-retest reliability, as assessed by Pearson correlations between CA-BAT scores in the first and second waves, was considerably worse for Study 4 than for Study 3 (Fig. 5b). With the full test length of 25 items the correlation between both sets of CA-BAT scores is only marginally statistically significant (*r*(40) = 0.30, 95% CI = [0.00, 0.55], *p* = 0.052), and the correlations are generally worse for shorter test lengths. These observed reliability coefficients are inconsistent with the simulated reliability coefficients, which predict performance broadly similar to Study 3.

#### Nomothetic span

Nomothetic span was investigated by computing correlations between CA-BAT scores and scores on three other measures: the melodic discrimination test, the temporal order discrimination test, and the Musical Training sub-scale of the Gold-MSI. Since the CA-BAT was administered in both waves it was possible to compute two correlations for each measure: one using CA-BAT scores from the same wave and one using CA-BAT scores from the other wave. The resulting correlations are plotted in Fig. 5c.

Contrary to Study 3, Fig. 5c exhibits no significant correlations between the Gold-MSI Musical Training sub-scale and the CA-BAT, irrespective of which wave the CA-BAT data come from. Correlations between CA-BAT scores and melodic discrimination scores reach broadly similar values at a test length of 25 (different waves, *r*(40) = 0.48, 95% CI = [0.20, 0.68], *p* = 0.001; same waves, *r*(183) = 0.45, 95% CI = [0.33, 0.56], *p* < 0.001). Correlations between CA-BAT scores and temporal order discrimination scores exhibit the largest dependency on testing session. When scores are compared across different testing sessions, the correlation only becomes statistically significant at a test length of 23 (*r*(40) = 0.31, 95% CI = [0.01, 0.56], *p* = 0.047, but when scores are compared from the same testing session, the correlation becomes statistically significant after 8 items (*r*(40) = 0.38, 95% CI = [0.09, 0.61], *p* = 0.013).

#### IRT parameter distributions

**Figure 6:**
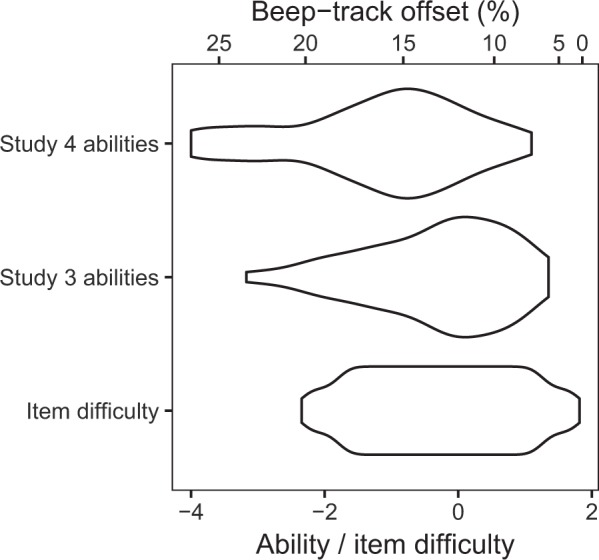
Person ability distributions for Studies 3 and 4, with the distribution of difficulties in the CA-BAT item bank plotted for comparison. Item difficulties are mapped to corresponding beep-track offsets for ‘ahead-of-the-beat’ items.

Figure 6 compares ability distributions and item difficulty distributions for the two validation studies (Study 3 and Study 4, taking ability estimates from the first waves). It can be seen that, while the ability distribution in Study 3 matches the item difficulty distribution fairly well, the ability distribution in Study 4 has a long tail of low abilities that are not matched by the item difficulty distribution. This means that the CA-BAT did not contain sufficiently easy items for these lowest-ability participants.

### Discussion

The purpose of this study was to investigate the performance of the CA-BAT when administered to online participants, as well as to investigate further the test’s nomothetic span. Similarly to Study 3, participants took the CA-BAT twice with approximately a week between testing sessions, as well as being administered two other listening tests: a melodic discrimination test and a temporal order discrimination test.

The results display a substantial decrease in test performance in the online setting. Though IRT-based reliability metrics (standard error of ability estimates, simulated test-retest reliability) only predicted a minor decrease in reliability, the empirical test-retest reliability estimates are much lower than observed in the laboratory-based Study 3, with the correlation between ability estimates across the two test waves only marginally significant after 25 items.

One contributor to this worsened performance may be lower participant abilities; in particular, several participants received ability estimates so low that even the easiest items in the CA-BAT item bank were too difficult for them. The resulting mismatch between ability and item difficulty impairs test reliability. However, IRT-based reliability metrics automatically account for this effect, and so the discrepancy between IRT-based and empirical reliability metrics remains unexplained.

An alternative hypothesis is that the online participants did not take participation seriously enough, and therefore displayed erratic and unpredictable behaviour when taking the CA-BAT. This hypothesis seems unlikely, however, when looking at the correlations between CA-BAT scores and other measures. In particular, correlations with melodic discrimination scores are statistically significant for both waves, and correlations with temporal order discrimination scores are significant when the two tests were administered in the same wave. It is strange that CA-BAT scores in the second wave should be better predicted by melodic discrimination scores in the first wave than by CA-BAT scores in the first wave, and this is not explained by participants not taking the test seriously.

A more plausible explanation for these results is that listening conditions have a particularly strong effect on CA-BAT performance, and while listening conditions tended to remain constant for an online participant within testing sessions, they tended to change between testing sessions. For example, in one session a participant might have worn headphones and sat in a quiet room; in the other session, the participant might have ignored the instructions to use headphones and sat in a public place. The CA-BAT and the temporal order discrimination test seem particularly likely to be vulnerable to changes in listening conditions, because they both rely on easily disrupted attention to fine perceptual detail. In contrast, the melodic discrimination test is likely to be less vulnerable to changes in listening conditions, because task difficulty stems less from perceptual features and more from memory-related features^21^. This would explain why correlations between CA-BAT scores and melodic discrimination scores were the most consistent of the correlations observed, and why CA-BAT and temporal order discrimination scores were highly correlated in the same wave but less correlated in different waves.

The implied lesson is that controlled experimental conditions are particularly important for the CA-BAT, and this constraint is easy to violate with an online study. Future work is required to see what measures could be used to improve the reliability of online CA-BAT administration. For example, it would be useful to guarantee that all participants use headphones, using techniques such as those suggested in ref.^58^.

## General Discussion

This research set out to apply modern psychometric techniques–IRT, adaptive testing, and automatic item generation–to the construction of an adaptive test of beat perception ability, the CA-BAT. The purpose of deploying these techniques was to gain a better understanding of the psychometric properties of the test paradigm, and thereby to produce improvements in test validity, reliability, and efficiency.

The development of this test involved a calibration phase and a validation phase. The calibration phase provided an opportunity to explore how structural features of CA-BAT items predict item difficulty. The results indicated that item difficulty could largely be predicted by the degree of beep-track displacement, with larger displacements yielding lower difficulty. Further improvements in predictive performance were achieved by taking into account the direction of beep-track displacement (behind-the-beat displacements are easier to detect than ahead-of-the-beat displacements) and individual differences between musical tracks. The effects of beep-track accuracy and musical track are consistent with the proposed item-processing model for the CA-BAT, and therefore contribute to the test’s construct representation and construct validity. The unexpected effect of beep-track displacement direction, meanwhile, suggests that there are aspects of the CA-BAT paradigm that are not yet fully understood. Future work should seek to reproduce and explore this effect.

The validation phase explored the CA-BAT’s performance in both laboratory and online testing scenarios. The results indicated that the full-length test performs well in the laboratory setting, achieving state-of-the-art test-retest reliability. In this context CA-BAT performance proved to be a significant predictor of several aspects of self-reported musical sophistication. Reliability and validity was considerably worse in the online setting. The results suggested that the CA-BAT is particularly affected by test-taking conditions, over which experimenters have little control in online contexts. This suggests that the CA-BAT is currently only suitable for laboratory use, unless ways are found to enforce greater homogeneity in online test-taking conditions.

Adaptive testing can theoretically provide substantial improvements in test efficiency, allowing test lengths to be drastically shortened without compromising test efficiency^19,32^. Such drastic effects seem not to have occurred in this study; the efficiency of the CA-BAT seems broadly similar to previous BAT implementations (e.g. comparing test-retest reliability with that reported in ref.^6^). This is likely to be due to inaccuracies in item difficulty estimates, which in turn cause inaccuracies in ability estimates. Achieving accurate item difficulty estimates is particularly difficult under automatic item generation, as item parameters are not calibrated individually but instead are estimated from a statistical model. The present study provides a useful starting point for such a statistical model, but clearly there is much room for improvement.

One way of improving the CA-BAT’s statistical model would be to explore different non-linear functions mapping from beep-track offset to item difficulty. The current mapping seems to work fairly well for the range of offsets employed in the present study, but provides unrealistic outcomes for very small offsets (as offset approaches zero, item difficulty should approach infinity, whereas the CA-BAT never predicts a difficulty greater than 2). A promising possibility would be to construct an improved mapping using non-linear item response modelling.

A second way of improving the CA-BAT’s statistical model would be to model musical track as a fixed effect, and thereby let musical track influence item difficulty estimates. Unfortunately in the present study we were unable to achieve model convergence with this approach. However, there are various options open for future work, such as collecting more response data and applying penalised regression techniques.

The lack of explicit manipulation of musical excerpts has implications for the construct validity of the CA-BAT. As item difficulty increases and the beep track moves closer to the true beat locations, the test will demand greater and greater precision in the listener’s beat inferences, but eventually it will also place demands on the precision of beep-track perception. At this point the test will become less of a beat perception test and more of a psychoacoustic test, comparable to the order discrimination test described previously. This problem could be addressed by incorporating musical excerpts where the beat is more ambiguous, ensuring that precision of beat inference remains the limiting factor in task performance. This possibility deserves to be explored in future work.

The adaptive testing algorithms used in the current implementation of the CA-BAT do not take into account imprecision in item parameter estimates. This is particularly problematic in the context of automatic item generation, since automatically generated parameter estimates are typically less reliable than traditional IRT parameter estimates. This functionality deserves to be incorporated into standard adaptive testing software packages such as catR^35^.

CA-BAT performance involves several cognitive processes, including perceptual encoding, beat induction, and working memory. Similar to previous work using the BAT paradigm^28^, we suggest that individual differences in CA-BAT ability primarily reflect individual differences in beat perception ability, at least in normal adult populations. However, it is important to verify this assumption in future work. It is also worth considering whether this assumption would hold for non-adult populations^15^ or populations with clinical disorders^27^. It seems likely that the influence of subsidiary abilities on CA-BAT performance would increase for populations with low levels of those abilities, as these abilities become limiting factors in task performance. In particular, the 2-AFC task introduces a reliance on working memory that could be problematic for memory-impaired populations. Generalising the CA-BAT to such populations must be done carefully, because the influence of these subsidiary abilities (e.g. working memory) would impact both construct validity (from CA-BAT scores measuring more than just beat perception) and test reliability (from subsidiary abilities breaking assumptions of the item response model).

Despite these limitations, the current CA-BAT should prove to be a useful tool for future research. Unlike pre-existing beat perception tests, the CA-BAT comes with a detailed psychometric model that can be used to substantiate the test’s construct validity. In the context of laboratory testing the CA-BAT achieves good reliability. Moreover, the modern psychometric techniques used in the CA-BAT allow for significant flexibility in test administration. Depending on time constraints and statistical power requirements, the researcher can refer to Fig. 4b and choose a balance between reliability and test length that best suits their current requirements. The IRT model can then generate precise estimates of measurement error which can then be explicitly accounted for in subsequent analyses.

The CA-BAT joins several other beat-processing measures in the literature, including the BAT^28^, A-BAT^29^, H-BAT^30^, and BAASTA^31^. These instruments have different advantages and disadvantages: some only test perception (CA-BAT, A-BAT), whereas some test perception and production (BAT, H-BAT, BAASTA); some are comprehensive but time-consuming (H-BAT, BAASTA), whereas others are faster but only measure limited aspects of beat processing (BAT, A-BAT, CA-BAT). Use of psychometric techniques varies between instruments, such as the use of IRT (CA-BAT) and adaptive testing (CA-BAT, A-BAT, H-BAT, BAASTA). Some instruments have also received more extensive validation than others. The CA-BAT contributes in providing a fast and well-validated test of an important aspect of beat perception, but it does not replace the H-BAT or BAASTA in assessing wider aspects of beat processing (e.g. duration discrimination, anisochrony detection, tapping). A promising route for future research is to extend our psychometric methods to other beat-processing tasks, such as those used in the H-BAT and BAASTA.

## Data Availability

Data and example code for fitting the CA-BAT item response models are available at 10.5281/zenodo.1211116. CA-BAT test materials (audio files and psychometric data) are available at 10.5281/zenodo.1210808. An implementation of the CA-BAT is available from the authors on request.
